# Application of Geographic Information Systems (GIS) in the Study of Prostate Cancer Disparities: A Systematic Review

**DOI:** 10.3390/cancers16152715

**Published:** 2024-07-30

**Authors:** Christiane J. El Khoury

**Affiliations:** 1 Program in Public Health, Renaissance School of Medicine at Stony Brook, Stony Brook, NY 11790, USA; christiane.elkhoury@stonybrook.edu; Tel.: +1-718-970-0177; 2 Department of Medical Oncology, The Sidney Kimmel Comprehensive Cancer Center at Thomas Jefferson University, Philadelphia, PA 19107, USA

**Keywords:** GIS, geographic, prostate cancer, disparities, systematic review

## Abstract

**Simple Summary:**

Prostate cancer (PCa) has significant disparities based on geography, affecting healthcare access and outcomes. This study reviews how Geographic Information Systems (GIS) are used to research these disparities. GIS helps visualize PCa incidence, survival, and mortality trends, but its application varies, leading to inconsistent results. The review followed Cochrane and PRISMA guidelines, analyzing 25 relevant studies. Most studies used GIS for mapping PCa data, geocoding, and spatial analysis to identify areas with poor PCa outcomes. However, inconsistencies in GIS methods and geographical scales used reduced the robustness of findings. The review suggests that better GIS techniques and interdisciplinary collaboration could improve the accuracy of PCa disparity research and support public health interventions.

**Abstract:**

**Introduction**: PCa is one of the cancers that exhibits the widest disparity gaps. Geographical place of residence has been shown to be associated with healthcare access/utilization and PCa outcomes. Geographical Information Systems (GIS) are widely being utilized for PCa disparities research, however, inconsistencies in their application exist. This systematic review will summarize GIS application within PCa disparities research, highlight gaps in the literature, and propose alternative approaches. **Methods**: This paper followed the methods of the Cochrane Collaboration and the criteria set of the Preferred Reporting Items for Systematic Reviews and Meta-Analyses (PRISMA). Articles published in peer-reviewed journals were searched through the PubMed, Embase, and Web of Science databases until December 2022. The main inclusion criteria were employing a GIS approach and examining a relationship between geographical components and PCa disparities. The main exclusion criteria were studies conducted outside the US and those that were not published in English. **Results**: A total of 25 articles were included; 23 focused on PCa measures as outcomes: incidence, survival, and mortality, while only 2 examined PCa management. GIS application in PCa disparities research was grouped into three main categories: mapping, processing, and analysis. GIS mapping allowed for the visualization of quantitative, qualitative, and temporal trends of PCa factors. GIS processing was mainly used for geocoding and smoothing of PCa rates. GIS analysis mainly served to evaluate global spatial autocorrelation and distribution of PCa cases, while local cluster identification techniques were mainly employed to identify locations with poorer PCa outcomes, soliciting public health interventions. **Discussion**: Varied GIS applications and methodologies have been used in researching PCa disparities. Multiple geographical scales were adopted, leading to variations in associations and outcomes. Geocoding quality varied considerably, leading to less robust findings. Limitations in cluster-detection approaches were identified, especially when variations were captured using the Spatial Scan Statistic. GIS approaches utilized in other diseases might be applied within PCa disparities research for more accurate inferences. A novel approach for GIS research in PCa disparities could be focusing more on geospatial disparities in procedure utilization especially when it comes to PCa screening techniques. **Conclusions**: This systematic review summarized and described the current state and trend of GIS application in PCa disparities research. Although GIS is of crucial importance when it comes to PCa disparities research, future studies should rely on more robust GIS techniques, carefully select the geographical scale studied, and partner with GIS scientists for more accurate inferences. Such interdisciplinary approaches have the potential to bridge the gaps between GIS and cancer prevention and control to further advance cancer equity.

## 1. Introduction

Prostate cancer (PCa) is the second leading cause of cancer death in American men and one of the cancers that exhibits the largest disparities [1,2]. There is a large literature documenting disparities in PCa outcomes that is robust across multiple regions and sociodemographic profiles [3]. African American (AA) men, on average, have a 78% higher incidence of developing PCa in their lifetime compared to Non-Hispanic White men (NHW) [1,2,4,5,6,7]. Further, AAs are also more likely to be diagnosed at a younger age, present with more aggressive disease, and possess a 2.3 times higher mortality rate than their NHW counterparts [4,5,6]. Hispanics and some Asian groups have lower PCa incidence; however, they tend to suffer from more advanced disease at diagnosis [5,6,8]. PCa disparities are not only present across racial/ethnic attributes, but they are also significantly associated with the geographical place of residence. Hispanics living in Mexico have a lower incidence of PCa than Hispanics living in the Caribbean [9], while Puerto Ricans living in Puerto Rico have a lower incidence than Puerto Ricans living in the mainland United States [10]. 

In 2019, a systematic review compiling results from 169 international studies presented substantial evidence that PCa outcomes and management varied according to the place of residence across different populations and geographies [11]. Although this review summarized the two most important drivers of PCa disparities, which were rurality and area deprivation, various geographical approaches were utilized across the studies, including multiple geographical scales and geospatial analyses, which created a wide heterogeneity for study comparison [11]. Other reviews have been published around nongeographical approaches for prostate cancer research, however, none have reviewed the utilization of geographic information systems (GIS) as tools to advance PCa disparities research [12,13,14,15]. In fact, Obertova and Afshar focused their reviews on inconsistencies of rural/urban designation and its utilization within PCa disparity research [13,14], while Gilbert discussed GIS approaches, however only focusing on the state of Florida [15]. 

According to the National Cancer Institute, health disparities research is a growing area in research, and tools to identify and eliminate disparities are growing and encouraged to identify pockets of disadvantage and map priority areas [16]. Geospatial analyses provide visual insights and substantial proof of the location of disparities and demonstrate their variability by adding a dynamic layer to traditional findings of disparities [17]. A new frontier of PCa research is the utilization of spatial approaches to identify focal points for interventions and resource mitigation and help outline underlying drivers of disparities [18]. 

Indeed, multiple approaches have been used to examine the association between geographical places of residence and PCa outcomes. Precisely, area-level characteristics and socioeconomic (SES) profiles have been linked to multiple disparities in PCa outcomes across various geographical scales such as county, census, census tracts, and others [19,20]. SES and demographics have also been linked to healthcare access and utilization of advanced PCa procedures [21,22,23]. Further, spatial approaches combine techniques from geography, epidemiology, and public health to better understand health needs and allocate resources [24]. This is especially relevant within the context of PCa disparities research, which calls for multidimensional approaches to advance cancer health equity and reduce the persisting gap in outcomes [1]. As such, GIS applications may help expose the determinants of local and sociodemographic disparities and provide information to improve health service delivery models, training for healthcare professionals, and overall health outcomes [25].

GIS is defined as any technology, software, or hardware that enables the processing, mapping, and analysis of geographical variables [26,27]. Geographic Information Systems (GIS) research in PCa has been developing throughout time and branched into multiple applications such as processing, mapping, and analysis [18]. The ultimate success of GIS is when data are transformed into a useful representation that provides disease insights [28]. Such a collaborative approach delivers prospects to examine associations and connections within health outcomes, the contextual environment, and social determinants of health to advance cancer-related equity research [29]. This allowed the advancement of such tools over time and the development of a field named Geographic Information Science (GIScience) [26], which examines the interdisciplinary collaborations aided by GIS to provide meaningful observations that have the potential to guide public health decision-making.

Furthermore, different geographical variables and various spatial scales have been adopted to conduct such analyses and provide valuable data for public health interventions [30]. As such, geographical analyses in PCa outcomes have moved from the simple stratification of rural/urban continuum to the computation of composite area deprivation indices within neighborhoods and utilization of GIS for cluster identification and prediction of poorer outcomes [31,32]. Those differences in approaches invite the need for methodological standardization when performing geospatial analyses to identify appropriate applications for Geographic Information Systems (GIS) in analyzing PCa disparities. 

The main goal of this comprehensive review is to compile a resource for researchers interested in conducting geographical analyses for PCa disparities. This systematic review aims to summarize the literature about geospatial disparities in PCa, describe the different GIS applications utilized in relating place of residence with disparities in PCa outcomes, and identify gaps in the literature. This review also identifies current limitations for GIS application in PCa research and proposes alternative approaches. As such, this review provides a comprehensive assessment of methods and a valuable resource for researchers joining the increasing trend of analyzing disparities from a geographical perspective. 

## 2. Methods

This paper follows the methods of the Cochrane Collaboration [33] and the criteria set of the Preferred Reporting Items for Systematic Reviews and Meta-Analyses (PRISMA) [34] to report systematic reviews and meta-analyses. Articles published in scholarly (peer-reviewed) journals in English were searched through the PubMed, EMBASE, and Web of Science databases until December 2022. The study has not been registered PROSPERO.

### 2.1. Search Method

The main search terms (i.e., MeSH terms and keywords) focus on (1) GIS (2) disparities and inequities (3) prostate neoplasm. Three main databases were researched PubMed, EMBASE, and Web of Science [35,36,37], and a detailed research strategy is included in Appendix A.

### 2.2. Article Selection

The population-intervention-comparison-setting (PICOS) method [33] was used to determine the eligibility of studies. In our reviewed articles, the participants were “adult men in the US diagnosed with PCa”, the intervention was the GIS approach, comparative groups were men from varying demographic/socio-economic backgrounds, outcomes were PCa incidence, mortality, and survival, and/or PCa management, and, finally, the studies included were observational. Eligible studies were all publications resulting from the database queries, referred publications known to the authors, and others gathered from the reference lists of the identified publications. Out of these eligible publications, an article selection process according to specified inclusion and exclusion criteria was conducted. Included articles were those employing a GIS approach for examining the relationship between geographical components and PCa disparities and/or inequities in the United States. Studies that examined disparities in PCa outcomes using geographical elements as independent variables were included, while studies conducted outside the US, those not published in the English language, and those that did not assess for a direct relationship between a geographical component and PCa disparities were excluded. No date restrictions were applied, and thus, the resulting articles were published through December 2022. The last date of search for relevant articles was 31 December 2022.

### 2.3. Study Management

**Figure 1:**
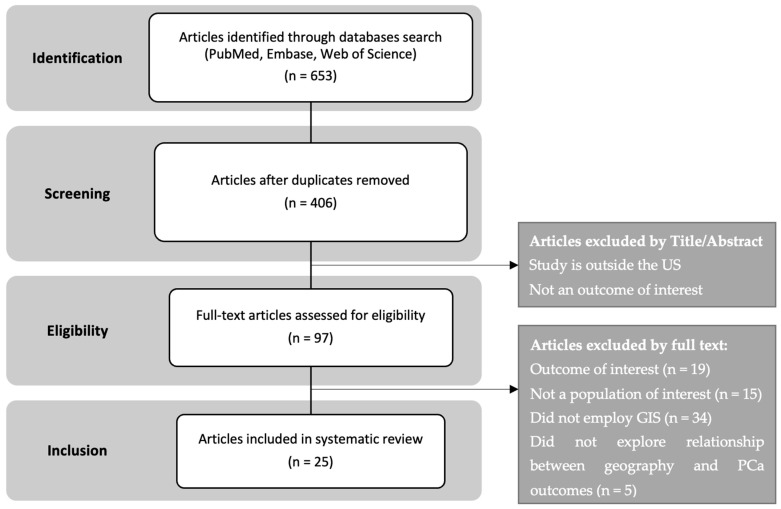
Article Selection Process.

All included articles were rightfully downloaded, managed, and screened using EndNote^®^. A total of 653 publications were deemed eligible, all published between 1998 to 2022, including 247 duplicates. Articles were screened for eligibility based on title and abstract, and 309 studies were disqualified due to the study setting not being in the US, not having an outcome of interest, and having no geographical component. After article selection according to the inclusion and exclusion criteria, 25 studies [38,39,40,41,42,43,44,45,46,47,48,49,50,51,52,53,54,55,56,57,58,59,60,61,62] met the requirements and were included in this review. Detailed reasons for full-text exclusions and the article selection process are represented in Figure 1. No potential biases were identified in the individual studies that met the inclusion criteria, as all resulting studies were evaluated based on reproducibility, methodological quality, and credibility.

Due to the nature of the research question that aims at reviewing discovered geographical disparities in PCa, publication bias may have arisen. Likely, studies with no significant findings for disparities were not published and, thus, included publications overrepresented disparities. However, the large population-based studies in this review tend to limit this potential overrepresentation. This review followed the PRISMA checklist for evidence-based reporting, and thus, principal summary measures were odds ratios, hazard ratios, relative risks, and differences in percentage, along with their respective *p*-values.

## 3. Results

In this systematic review, a total of 25 studies were included, published from 2002 to 2022; those studies are detailed in Table 1. A total of six studies examined disparities in late-stage PC, six in incidence, four in mortality and survival, three in incidence, grade, and stage simultaneously, two in mortality-to-incidence (MIR) ratio, and two in PCa management (Table 1). GIS applications were grouped into three main application purposes: “mapping”, “processing”, and “analysis” (Table 1). A summary of the key findings from these studies is found in Box 1 and Box 2.

Box 1 **Summary of Key Findings Related to PCa Disparities in GIS Studies.**

**GIS Findings for Disparities in Prostate Cancer Incidence**

- Higher PCa incidence was frequently associated with better socioeconomic status (SES) at the census-tract level, particularly in non-Hispanic Whites (NHWs) compared to African Americans (AAs).
- Urban residence and higher household income were linked to an increased likelihood of PCa diagnosis, suggesting enhanced healthcare access in these areas.
- The spatial variations in PCa incidence were influenced by factors such as income and education and comorbidities like diabetes and obesity.

**GIS Findings for Disparities in Prostate Cancer Stage and Grade at Diagnosis**

- Disparities in late-stage diagnosis were associated with lower SES, particularly in counties with lower income and education levels.
- Missing stage and grade information served as proxies for worse outcomes and were more common in areas with higher SES, suggesting discrepancies in data collection and reporting.
- Temporal analysis revealed that disparities in late-stage PCa diagnosis have declined over time, influenced by changes in PSA screening recommendations.

**GIS Findings for Disparities in Prostate Cancer Mortality and Survival**

- Geographical clusters of higher mortality rates were identified, with some areas showing significant disparities between racial groups.
- Survival rates varied significantly based on place of residence, with SES factors partially explaining these differences.
- Mapping studies highlighted that rural areas and those with higher poverty rates exhibited poorer PCa survival outcomes.

**GIS Findings for Disparities in Prostate Cancer Management**

- Disparities in PCa management were examined in two studies, with GIS mapping showing that treatment modalities were concentrated in urban areas.
- Travel distance impacted the likelihood of receiving certain treatments, with longer distances associated with a decreased probability of intervention.

GIS: Geographic Information Systems.

Box 2 **Summary of Key Findings from the Application of GIS in PCa Disparities Research.**

**Application of GIS in PCa Disparities Research: Mapping**

- GIS techniques were predominantly employed for mapping and visualization, translating PCa data into geographical polygons to provide a cartographic representation of PCa rates and zones of disparity.
- Mapping studies commonly used various geographical scales such as counties, census tracts, zip codes, neighborhoods, and census block groups.
- Visual mapping helped identify areas with higher PCa incidence and poorer outcomes, aiding in targeting further analysis and public health interventions.

**Application of GIS in PCa Disparities Research: Processing**

- Geocoding and smoothing were key GIS processing techniques used to prepare PCa data for analysis.
- Geocoding converted addresses into geographical coordinates, facilitating the visualization of individual-level data at various scales.
- Smoothing techniques like binomial kriging and spatial empirical Bayesian smoothing were used to reduce noise and provide clearer spatial patterns in the data.

**Application of GIS in PCa Disparities Research: Analysis**

- Spatial analysis methods identified geographic associations with PCa outcomes, utilizing techniques like global spatial autocorrelation and cluster identification.
- Global spatial autocorrelation assessed the overall geographical variability and clustering in PCa data.
- Cluster identification techniques such as the Spatial Scan Statistic, Getis-Ord-Gi, and local Moran’s I highlighted areas with significant PCa disparities, aiding in prioritizing public health interventions.
- A geographically weighted regression model was employed to examine spatially varying associations between predictors and PCa outcomes, highlighting areas where risk factors had a stronger influence.

GIS: Geographic Information Systems.

## 4. Summary of PCa Disparities Findings in GIS Studies 

### 4.1. GIS Studies That Examined Disparities in PCa Incidence

GIS studies examining disparities in PCa mainly shared a common purpose of identifying locations of higher-than-expected incidence and examining their associations with contextual factors. For example, in Connecticut and Massachusetts, clusters of high PCa incidence were characterized by a better census-tract-level SES (less than 12 years schooling rate, below the poverty rate, renter-occupied dwellings rate, unemployment rates) mainly in NHWs as compared to AAs [40]. Similarly, in Virginia, higher household income and urban residence increased the likelihood of diagnosis, suggesting that better census-tract SES enhances healthcare access, especially for PCa screening [41]. Furthermore, residing in urban census tracts was associated with early-stage diagnosis in a multi-state study conducted in Alabama, Tennessee, Georgia, and Florida [48]. Also in Georgia, Wagner et al. identified clusters of high PCa incidence that slightly differed in locations upon racial stratification, suggesting the involvement of environmental predictors [53]. A novel approach was adopted by Gregorio et al., as they demonstrated that the “detection effect” through adjusting for colorectal cancer screening accounted for all significant spatial variations in PCa incidence [54]. In Pennsylvania, the temporal decline in PCa incidence from 2000 to 2011 was suggested to illustrate the effect of the variation in PSA screening recommendations. Most notably, age at diagnosis was significantly younger in AAs as compared to NHWs, calling for increased attention in metropolitan Philadelphia areas where AAs are concentrated [56]. Mapping of PCa incidence in Alabama counties against rates of diabetes, obesity, education, and poverty, suggested an apparent association with those factors [61]. Accordingly, GIS studies examining disparities in PCa incidence suggested that higher PCa incidence may be associated with area-level racial composition, rurality, income, poverty, education, unemployment, percent renter-occupied dwellings, access to screening, and other chronic comorbidities.

### 4.2. GIS Studies That Examined Disparities in PCa Grade and Stage at Diagnosis

Having a “missing” stage and/or grade information from the tumor registry was utilized as a proxy for possible worse PCa outcomes. For example, Klassen et al. examined the relationship between missing stage and/or grade and area-level SES. As such, clusters of having a missing PCa stage or grade from the Maryland Tumor Registry were identified. Having a missing stage was associated with higher county-level household income, while having a missing grade was associated with higher census block-group household income [39]. In Florida, northern and central counties exhibited the greatest racial disparities in late-stage PCa, which was associated with lower census-tract income and lower college education [43]. Additionally, the late-stage proportions decreased significantly from 1981 to 2007, however, the rate of decline varied greatly based on county location and racial groups [49]. This variation was suggested to be related to geographical disparities in the implementation of Prostate Specific Antigen (PSA) screening [52]. Upon racial stratification, more counties exhibited higher proportions of late-stage diagnosis in AAs versus NHWs. Associations were also detected on the census tract level as higher census tract income was protective, while the presence of farmhouses increased the likelihood of a later-stage diagnosis [49,55]. Moreover, a side-by-side mapping comparison of late-stage odds ratios (ORs) with comorbidities, income, and smoking rates at the county level suggested that those could be associated with a later-stage diagnosis [55]. Thus, in addition to establishing relationships between later-stage diagnosis and poorer area-level SES, the temporal factor was also important to account for within this context, especially when it comes to varying PCa screening recommendations and clinical practices [63].

### 4.3. GIS Studies That Examined Disparities in PCa Mortality and Survival

Using national data of PCa patients from 1970 to 1989, five national clusters of higher mortality in NHWs and three in AAs were detected; however, those could not be attributable to the selected county-level SES variables, which included education and agricultural employment [38]. Identified geographical clusters of poorer PCa survival in Connecticut significantly diminished when individual-level variables representing age, race, and tumor severity (stage and grade) were accounted for, suggesting that survival only varies in part according to the place of residence and other area-level factors might be predictors [42]. In Texas, counties with statistically significant excess mortality rates were found to be concentrated in the center of the state for multiple racial subgroups in a spatial and temporal analysis over a 22-year study period [44]. Meliker et al. identified survival disparities across the state of Michigan. Existing disparities identified at larger geographical scales, such as Federal House Legislative Districts (FHLD), diminished and sometimes disappeared upon examination on smaller geographical scales, such as State House Legislative Districts (SHLD) and neighborhoods. This was attributed to the fact that, in smaller areas, the population at risk is more uniform in terms of modifiable SES, risk factors, and proximity to cancer screening [46]. In South Carolina, Hebert et al. mapped racially stratified MIRs across eight Department of Health and Environmental Control (DHEC) regions. Visualization on mapping presented striking differences between AAs and NHWs allowing for the localization of areas with the widest disparity gaps. MIR was also mapped per Zip Code Tabulation Area (ZCTA) in South Carolina for US Veterans, where metropolitan MIR was found to be higher than non-metropolitan MIR, and two clusters of higher-than-expected MIRs were detected in the upstate region. In contrast to Hebert’s finding above, Georgantopoulos et al. found that AAs had a lower MIR than NHWs, suggesting that Veterans exhibit a more uniform population for comparison and that factors causing such disparities are likely modifiable and related to healthcare access and SES [59]. Finally, PCa mortality hotspots were heavily concentrated in three major areas in Georgia. “Hotspot counties” generally had a higher proportion of AAs, older adult population, greater poverty, and more rurality [60]. Although area-level SES was shown to be associated with poorer PCa survival, including facility-level characteristics within GIS studies, as in Georgantopoulos’s study (2021), provided an additional layer for examining racial disparities in PCa.

### 4.4. GIS Studies That Examined Disparities in PCa Management

Only two studies examined disparities in PCa management. Those mainly employed GIS mapping to identify visual associations between zip-code level factors and PCa treatment. Single-institution data were used to relate Stereotactic Body Radiation Therapy (SBRT) with zip-code level characteristics. The geospatial distance between the patient’s zip code and the facility was calculated and the geographical reach of the institution was assessed by examining the SES status for each zip code. Travel distance did not prevent the uptake of prostate SBRT in AAs, elderly, or rural localized PCa cases [58]. A national GIS study examined disparities in PCa management using the National Medicare Database, where PCa modalities were mapped across PCa cases by county. Multivariate regression identified that practitioners of more novel modalities (i.e., SBRT and proton therapy) were mainly concentrated in more urban zip codes, while greater distance was associated with a significantly decreased probability of treatment (IMRT—3.8% per 10 miles; prostatectomy—2.1%; brachytherapy—2%; proton therapy—1.6%; and SBRT—1.1%) [62].

## 5. Application of GIS in PCa Disparities Research

**Figure 2:**
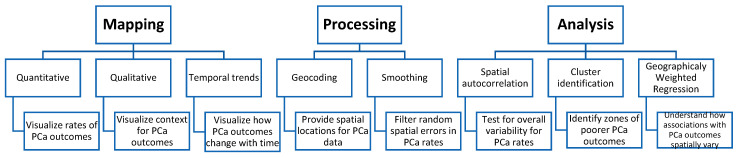
GIS application in Prostate Cancer (PCa) Disparities Research.

All included studies shared a mutual rationale for GIS employment, which was to identify geographic regions with the highest burden of PCa so that public health interventions could be prioritized. In this systematic review, three main purposes were identified for utilizing a GIS approach in studying PCa disparities: mapping, processing, and analysis. Mapping was employed in 24 studies, analysis in 16, and processing in 14 (Table 1). They are described below and are represented in Figure 2.

## 6. Application of GIS in PCa Disparities Research: Mapping

All but one study [45] employed GIS techniques for mapping/visualization where PCa data were mostly translated into polygons of PCa measured in a certain geographical unit. The main purpose of creating maps was to provide a cartogenic representation of PCa rates and zones where poorer outcomes or higher disparities exist. Multiple software was utilized for mapping; however, ArcGIS remained the most utilized as it was employed by 9 out of the 23 studies included, and it is considered by many as the industry standard [64,65].

### 6.1. Mapping a Snapshot in Time: Qualitative and Quantitative Data

All studies presented maps with a single snapshot in time, mostly translating points to polygons, as point data were aggregated to a certain designated geographical scale. The most common scale for mapping was by county, present in 12 studies. Remaining mapping was performed on the level of the census tract (in 3 studies [40,41,48]), zip codes (2 studies [58,59]), FHLD/SHLD/Neighborhoods (1 study [46]), DHEC (1 study [47]), and census block group (1 study [39]). For example, after acquiring individual-level data from the Virginia Cancer Registry, Oliver et al. geocoded data to the street level and assigned a census tract and a county for each case. As such, maps were reproduced, displaying county-level and census tract-level PCa incidence. Such mapping helped to visually identify how disease rates changed from one zone to another. Consequently, PCa incidence was found to be the highest in the eastern and central portions of Virginia [41]. Such visual indicators can be the source of identifying locations where further analysis of contextual factors might be warranted.

**Figure 3:**
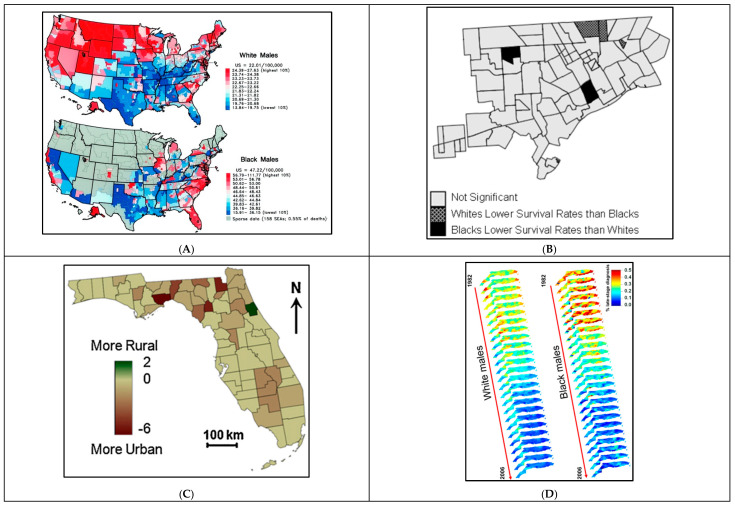
Examples of different types of mapping utilized in PCa disparities research. (**A**). Quantitative Mapping: PCa mortality rates. Prostate cancer mortality rates among White males (upper panel) and Black males (lower panel) by state economic area, 1970–1994 [38]. (**B**). Qualitative Mapping: Presence/absence of disparities. Significant racial disparities in prostate cancer survival in neighborhoods in Detroit, Michigan, 1990–1998 [46]. (**C**). Qualitative Mapping: rural/urban counties. Maps of rural/urban continuum codes for Florida counties over the period of 1993–2003 [51]. (**D**). Trends in time: Three-Dimensional mapping. A 3D representation of 25 maps of county-level proportions of late-stage PCa in Florida from 1982 to 2006 [49].

Furthermore, both qualitative and quantitative PCa-related variables were represented (Figure 3). Eleven studies had both quantitative and qualitative maps, while eight had only quantitative and six only qualitative. Qualitative mapping showed the spatial distribution of categorical or nominal data, such as rural/urban counties, or the presence or absence of certain outcomes, such as zones presenting significant disparities or clusters of a concentrated outcome (Figure 3). Conversely, quantitative mapping presented the spatial distribution of numeral data, as most of those represented PCa rates, either for incidence, late stage, or mortality (Table 1). This kind of mapping was mainly used to identify locations with worse PCa outcomes or higher concentrations of disease. For example, Jemal et al. mapped PCa mortality rates per county relying on the national cancer registry data. This approach was useful for identifying and visualizing counties with higher PCa mortality by comparing mortality rates across US counties (Figure 3A).

One of the uses for qualitative mapping was to illustrate the presence (or absence) of objective differences and/or inequities between specific subpopulations of interest. For example, Meliker et al. mapped locations with significant racial disparities in PCa survival to highlight areas of unequal PCa outcomes (Figure 3B) [46]. Qualitative mapping was also utilized to map contextual variables that help in understanding spatial circumstances under which PCa outcomes may be affected. This was especially valuable when qualitative information was visualized in parallel to PCa outcomes. For instance, Goovaerts et al. produced a qualitative map of rural/urban counties to obtain a visual representation of the associations between rural/urban places of residence and late-stage diagnosis (Figure 3C) [52].

### 6.2. Mapping Trends Overtime

Although mapping either qualitative or quantitative data in a time snapshot offers insightful visualization, including a temporal dimension ensures a more complete geographical analysis across the period studied. Hsu et al. included a temporal element in their mapping by reproducing maps showing excess PCa mortality across different time frames [44]. The inclusion of the temporal dimension allowed them to not only identify geographical clusters of worse PCa mortality but also to examine whether those clusters persisted over time. As such, their mapping identified three specific counties where excess mortality among Hispanics has been consistently present for over 19 years, calling on public health policymakers to prioritize those areas based on spatiotemporal evidence [44]. Gooavert et al. [49,51] furthered the inclusion of the temporal dimensions through 3D mapping of PCa incidence and late-stage diagnosis [51]. Their three-dimensional model was created using SGeMS, Stanford Geostatistical Modeling Software, where proportions of late-stage PCa were calculated over a 3-year moving window from 1982 to 2006 (Figure 3D). This mapping approach allowed the examination of how rates of late-stage disease responded to the 1990s introduction of Prostate Specific Antigen (PSA) testing, a blood test that facilitated PCa detection and early diagnosis. As such, including a temporal dimension while mapping PCa outcomes makes it easier to comprehend spatiotemporal relationships, especially as significant approaches that affect clinical guidelines and health outcomes are continuously developing in PCa. 

## 7. Application of GIS in PCa Disparities Research: Processing

Processing spatial data was mainly performed in 14 studies to prepare data for subsequent analyses and was grouped into geocoding and smoothing. (Table 1). Eight studies mentioned geocoding their data, six studies employed smoothing techniques, and two studies employed both (Table 1).

### 7.1. GIS Processing: Geocoding

Geocoding allowed the provision of geographical coordinates for participants’ addresses that were later used for mapping and allowed for individual-level variables to be represented on a location basis. Accordingly, addresses of PCa cases were geocoded into a specific location to facilitate spatial recognition patterns and allow for observational inferences. For example, Oliver et al. geocoded their PCa cases to the census tract using exact patient addresses, which allowed examining associations between high PCa incidence and census-tract-level SES [41]. Another application of GIS processing is the transformation of certain point variables to aggregates, which provides variable information for multiple geographical scales. For instance, Xiao et al. employed GIS processing to transform available latitude and longitudinal data into values per county to examine how county-level environmental factors affect PCa outcomes. In this case, geocoding assisted in preparing environmental data for county-level mapping and analysis by testing the relationship between county-level environmental factors and PCa stage/grade [43]. As such, GIS processing allows for scale transformation and the obtention of variables to the desired level of aggregation to be able to draw inferences between area-level characteristics and PCa outcomes. 

Although geocoding enabled scaled visualization and data transformation, geocoding percentage, describing the successful conversion of addresses into a specific location, varied between studies. Half of the studies that mentioned geocoding did not report the percentage of successful geocoding (Table 1). The geocoding success rate in the remaining half ranged between a low of 74% [41] to a high of 100% [47]. Notably, geocoding success increased with the increasing size of the geographical scale as it moved from 74% upon geocoding to the census tract to 100% upon geocoding to the county [41].

### 7.2. GIS Processing: Smoothing

**Figure 4:**
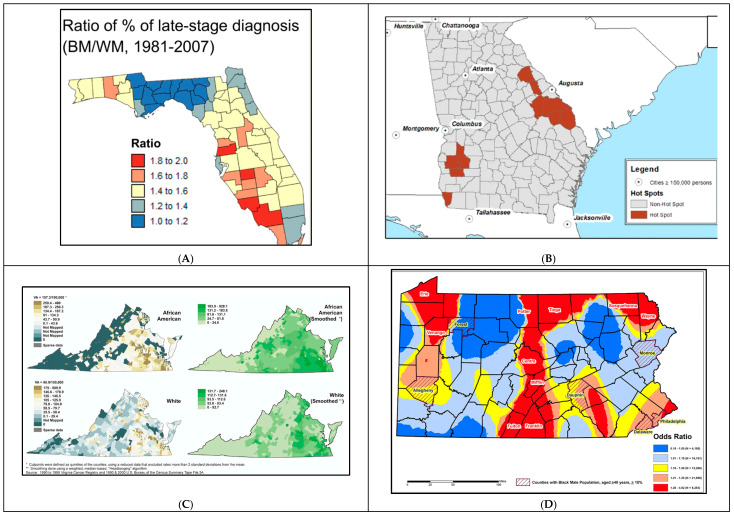
Application of smoothing techniques in GIS studies examining PCa disparities. (**A**). Time-average proportions of prostate cancer late-stage diagnosis: Black Male/White Male represents disparities in late-stage diagnosis between AAs and NHWs [49]. (**B**). Prostate Cancer Mortality Hotspots in Georgia: Hotspots were based within the fifth quintile of smoothed spatial Empirical Bayes (EB) of PCa mortality rates [60]. (**C**). Annualized age-adjusted prostate cancer incidence rates per 100,000 population (left) by Census Tract, 1990–1999. Smoothed rates (right). African Americans (top) and Whites (bottom). A total of 74% of all cases were geocoded to the census tract [41]. (**D**). Spatial variation on the local risk of highly aggressive prostate cancer in Black compared to White men diagnosed with prostate cancer, Pennsylvania 2004–2014 [57].

Data smoothing created an approximation function intended to capture patterns in the dataset and was mainly employed to reduce noise in the data by providing smoothed estimates (Figure 4). Goovaerts et al. performed binomial kriging to smooth rates of late-stage PCa to obtain smoother maps for late-stage diagnosis rates, while Moore et al. (2022) employed the spatial empirical Bayesian smoothing (SEBS) method to smooth mortality rates [60]. In both cases, smoothing was mainly utilized to approximate rate data and filter random noise so that clearer spatial patterns could be observed. 

An additional reason binomial kriging is performed is to replace missing values from the years where no PCa cases were diagnosed within specific locations in Florida [49]. Binomial kriging provided a measure of reliability called the kriging variance that allowed capitalizing on spatial autocorrelation and neighboring geographical units. This was followed by a sensitivity analysis, which showed that kriging-based noise-filtering improved the fit of the joinpoint regression models (i.e., lower residual variability) compared to the modeling of raw rates. In this case, noise-filtered data also helped in providing a clearer detection of the variation in county-level late-stage diagnosis rates across racial groups and study periods (Figure 4A) [49].

Moore et al. applied the SBES method to smooth PCa mortality rates and group them into quintiles. This distribution allowed for quintile-based quantitative mapping to identify and represent counties belonging to the poorest quintiles of PCa mortality (Figure 4B). Such an initial approach only provided information on how counties compare in terms of PCa outcomes without identifying clusters or hotspots of concern [60]. On another hand, a weighted two-dimensional smoothing algorithm, called Headbanging, was performed on PCa incidence rates in Virginia (Figure 4C). This allowed for smoother mapping of PCa outcomes, allowing patterns to emerge from the data [41]. Lastly, the Inverse Distance Weighting (IDW) interpolation technique was performed to provide smoothed GIS mapping based on local odds ratios of highly aggressive PCa [57]. This technique created continuous and smoothed surfaces for the entire state of Pennsylvania based on the respondents’ addresses. This allowed the visualization of spatial patterns of the explanatory effect of the variable “race” as smoothed rates were racially stratified (Figure 4D).

## 8. Application of GIS in PCa Disparities Research: Spatial Analysis

Although mapping and processing may produce key visual insights, spatial associations can be examined by utilizing specific GIS analysis methods. In this systematic review, 16 studies applied GIS analysis to spatially analyze and interpret associations with PCa outcomes. Of those, 4 performed global spatial autocorrelation, 15 included a cluster identification approach, and 1 study employed a geographically weighted regression (Table 1).

### 8.1. GIS Analysis: Identification of Spatial Autocorrelation

Spatial autocorrelation is the term used to describe the presence of systematic spatial variation in a variable, and it is the tendency for areas or sites that are close together to have similar values [66]. As Waldo Tobler’s first law of geography states, “Everything is related to everything else. But near things are more related than distant things” [67]. This was used as a key concept in geospatial research as it laid the rationale of spatial autocorrelation methods that test whether geographically closer zones have more of the same health outcome profiles. Spatial autocorrelation indicated the presence of clustering or dispersion in a map; as such, examining the global spatial autocorrelation was used as an initial step for assessing overall geographical variability in the study area and was performed in 4 out of the 25 studies included (Table 1). Three spatial tests were utilized to assess for global autocorrelation: the Global Moran’s I, Cuzick–Edwards’ k-NN, and Tango’s Maximized Excess Events Test (MEET) (Table 1). 

Data from the Pennsylvania Cancer Registry were used to test for significant global autocorrelation using Global Moran’s I. The Global Moran’s I statistics with 95% confidence intervals were calculated for each of the four time periods studied (2000–2002, 2003–2005, 2006–2008, and 2009–2011) and resulted in a non-significant negative value, indicating a non-significant negative spatial autocorrelation or a dispersed pattern in the data. As such, the authors’ interpretation included the presence of heterogenous dispersion of PCa incidence across counties, which was also apparent in the quantitative mapping [56]. Similarly, a non-significant Global Moran’s I (*p* = 0.08) was also obtained upon testing for spatial autocorrelation of MIRs in South Carolina [59]. Despite the lack of statistically significant global heterogeneity, subsequent local cluster identification techniques detected two significant clusters of higher-than-expected MIRs [59]. Although examining global spatial autocorrelation was mainly utilized to test for general dispersion or clustering of the whole area of study, this approach did not eliminate the presence or absence of local PCa clusters.

A comparative study was performed to compare three different global spatial clustering techniques, utilized commonly in GIS research, to test for clustering in PCa stage and grade: Cuzick–Edwards’ k-NN, Global Moran’s I, and Tango’s Maximized Excess Events Test (MEET) [45]. Cuzick–Edwards’ k-NN and Moran’s I were found to be very sensitive to the population’s density, while MEET turned out to be the simplest to use, as density does not need to be specified for the test. For the stage at diagnosis, all three models showed a reduction in clustering upon individual and area-level adjustments; however, some residual clustering remained. This study showed that, in addition to testing for global dispersion, those three global clustering techniques can be applied to check for residual clustering, especially after adjusting for individual and area-level variables [45]. All in all, assessing for global clustering allows for identifying dispersion in overall PCa outcomes within spatial data. This initial step was important to understand the level of geographical heterogeneity of the PCa measure in question and elicited the need to adjust for underlying factors.

### 8.2. GIS Analysis: Cluster Identification

**Figure 5:**
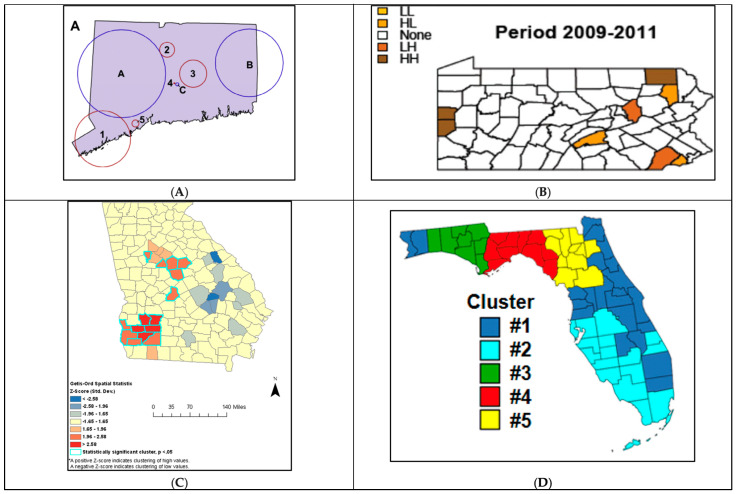
Application of GIS Analysis in PCa Disparities Research. (**A**). Geographic incidence clusters of invasive prostate cancer adjusted for age at the time of diagnosis Connecticut, 1994–1998: adjusted for age at time of diagnosis (circle A), age and race (circle B), and age and poverty level (circle C) [54]. (**B**). LISA cluster maps for White men in Pennsylvania (2009–2011) [56]. (**C**). Getis-Ord Gi * statistic for hotspot analysis of PCa incidence for both races by county, 1998–2008, Georgia [53]. (**D**). Results of spatially weighted classification of 67 counties in Florida: grouping of counties based on the similarity of their temporal trends in proportions of late-stage diagnosis and their geographical proximity [51].

In addition to assessing for global spatial autocorrelation, GIS was utilized to identify clusters of concern in 14 studies, as this was often performed with the aim of identifying and prioritizing zones for public health interventions and/or locations that elicit further analyses (Table 1). Methods of cluster detection varied (Figure 5) as eight studies employed the Spatial Scan Statistic, two the local Moran’s I, two utilized a spatially weighted hierarchical cluster analysis, one performed a hotspot analysis coupled with the Spatial Scan Statistic, and another coupled with the local Moran’s I test (Table 1).

The Spatial Scan Statistic developed by Kulldorff [68] was commonly used to identify whether PCa outcomes were geographically randomly distributed or whether clusters were present. Within these studies, SatScan software was utilized to generate ellipses and/or circles of varying sizes and evaluate observed versus expected rate ratios (risk within vs. outside the circles) to identify statistically significant “clusters” of disease rates [68]. Variations in the utilization of Kulldorff’s Spatial Scan Statistic are identified and described in Table 2. Six studies relied on circular scanning windows, one on both circular and elliptical, and two did not mention the scanning window shape employed. Variations in scanning window size also occurred, which were mostly dependent on the size of the population at risk (four studies) and on the study period (one study). Furthermore, the cluster delimitation approach was different among studies, as five studies did not rely on geopolitical boundaries for cluster formation, while three based their clusters on county and census tract boundaries (Table 2).

Although all studies utilizing the Spatial Scan Statistic shared a similar purpose, several rationales were employed. Some studies relied on racially stratifying cluster identification to highlight racial disparities in PCa outcomes. For example, four clusters of higher PCa incidence were detected in NHW, while two clusters were detected in AA within the states of Connecticut and Massachusetts between 1994 and 1998 (Figure 5A) [40]. Other studies attempted to understand the underlying factors behind cluster formation by testing whether identified clusters remained after adjusting for designated factors. As an example, the number of significant clusters diminished when adjusting for individual-level variables such as race, age, and year and census-tract level SES. This approach explained the potential variables affecting cluster formation as older age, Black race, and higher county-level income increased the likelihood of missing stage while older age and higher block-group income increased the likelihood of missing grade [39]. Similarly, the number of clusters of poorer PCa survival decreased in Connecticut upon adjusting for disease severity. However, the fact that some of those clusters remained demonstrated that additional factors not accounted for in the study, were contributing to worse PCa prognosis [42]. Another approach for employing cluster identification is to profile the SES characteristics of the identified clusters in order to understand the relationship between poorer outcomes and area-level variables within those specific geographical boundaries. For example, Altekruse et al. focused on gathering clusters of higher PCa incidence to examine the relationship between high incidence within those boundaries and area-level SES utilizing the Pearson correlation test [48]. This resulted in significant associations between a higher relative risk of localized PCa and urban locations as well as higher AA proportions [48].

The Getis-Ord-Gi technique developed by Getis and Ord in 1992 was also used to identify hotspots of concentrated disease outcomes [69]. In contrast to clusters identified by the Spatial Scan Statistic, this approach mainly identified “cooler” or “hotter” zones of the designated outcome in question. For example, in the state of Georgia, Wagner et al. analyzed county-level hotspots of PCa incidence with the Getis-Ord-Gi statistic and identified census-tract level clusters using the Spatial Scan Statistic. The rationale behind this dual cluster identification approach was primarily to identify counties with the highest PCa incidence and delineate clusters of higher incidence within smaller geographical areas [53]. Another county-level hotspot analysis was performed in Georgia to detect counties with the highest PCa mortality (Figure 5C). The analysis was then racially stratified to compare racial disparities in PCa mortality. The identified hotspot counties were then analyzed for SES characteristics and found to have a higher AA proportion and lower median household income when compared with non-hotspot counties [60].

Furthermore, three studies employed the local Moran’s I to identify Local Indicators of Spatial Autocorrelations (LISA) (Table 1). LISA was used to identify significant clusters of Pennsylvania counties with either higher or lower PCa incidence as well as counties that differed significantly from their neighboring counties, representing either a “high-low” or “low-high” geographical cluster (Figure 5). In addition to identifying low and high-incidence counties, LISA provided information on how a specific location compared with its surroundings (Figure 5B). The analysis was repeated for four different time periods to understand the temporal variation of identified clusters [56]. Lastly, two studies employed the spatially weighted hierarchical cluster analysis using Ward’s minimum variance to group counties that have similar temporal trends of late-stage incidence rates in the state of Florida. This was mainly performed to examine the temporal and spatial clustering of late-stage proportions, especially since screening recommendations were introduced during the study period (Figure 5D) [51,52]. 

### 8.3. GIS Analysis: Geographically Weighted Regression (GWR)

Only a single study employed GWR (Table 1), which provided a spatial dimension to traditional measures of associations. A geographically weighted local logistic regression model was used to investigate how the covariate effects on PCa outcome changed spatially by considering spatial dependence. In fact, higher weight was assigned to cases that were geographically closer to each other to account for spatial dependence. This method was mainly applied to represent how associations between predictors and PCa outcomes vary geographically. For example, Goovaerts et al. identified specific areas where the risk of advanced PCa is more sensitive to the census-tract median household income [55]. 

## 9. Discussion

This systematic review is the first to comprehensively summarize GIS applications in prostate cancer (PCa) disparities research. Unlike previous reviews that focused on geographical variability in PCa outcomes and associations with predictors, this review emphasizes the utility of GIS [11,12,14,18]. GIS’s interdisciplinary approach is crucial for addressing disparities in PCa outcomes [6,70].

### 9.1. Main Themes and Findings

GIS applications in PCa disparities research fall into three main themes: mapping, processing, and analysis. Most studies (23 out of 25) utilized GIS to examine PCa incidence, mortality, and survival rather than treatment and management. The primary rationale was to visualize and statistically identify geographical areas with poorer PCa outcomes, aiding in policy and public health intervention prioritization. Policymakers could also benefit from identifying disparities in healthcare access, as disparities in procedure utilization and PCa management contribute to worse outcomes [20,21,22,71]. A clear limitation in examining PCa management outcomes in GIS research is the databases used. Including databases with procedure information, such as SEER-Medicare [72] or SPARCS [73], could enhance GIS research by visualizing healthcare access disparities and associating them with outcomes. Despite this, cancer registry data linked to census data proved valuable for examining PCa outcomes and area-level characteristics (Table 1). 

### 9.2. Specific GIS Applications in PCa Management

Two studies focused on PCa management, using GIS for mapping and regression analyses to explore the relationship between radiation therapy uptake, travel distance, and socioeconomic status (SES) [58,62]. Aghdam et al. mapped SES clusters of patients receiving radiation therapy [58], while Tang et al. mapped PCa treatment modalities by county [62]. Other studies also examined the impact of travel distance on treatment utilization, finding that longer distances were associated with lower radiation therapy likelihood [74,75] and increased advanced-stage PCa rates among African Americans [76]. Dobbs et al. used Google Distance Matrix API to calculate transit times and their impact on clinic absenteeism, finding driving distance inversely associated with missed appointments [77]. This approach could help study the impact of distance and time on healthcare access among PCa patients. Combining procedure uptake information with analytical GIS approaches could provide insights into healthcare access for PCa patients. Such approaches have been used to study spatial variation and identify clusters in other diseases, such as malignancies and vaccine uptake [78,79,80]. For example, Zahnd et al. performed hotspot analysis and spatial lag models to detect low mammography access clusters and identify associated sociodemographic factors [80]. Translating these approaches to PCa procedure uptake, such as multiparametric MRI for advanced diagnosis and detection, could advance the understanding of PCa disparities. This is crucial as PCa is a screenable and highly curable disease when appropriate screening and management are undertaken.

### 9.3. Multilevel Analyses in GIS Research

Four studies successfully integrated GIS with multilevel analyses, an essential approach given the complex relationship between race/ethnicity and area-level SES in PCa disparities [39,41,43,50]. Klassen et al. identified high PCa grade and stage clusters and evaluated variability before and after adjusting for census-level characteristics [39]. This approach helps determine the contribution of multileveled factors to spatial clusters and identifies areas for additional localized investigations. Similarly, Altekruse’s study further examined identified clusters for local associations with area-level factors [48].

### 9.4. Limitations and Recommendations for GIS Mapping, Processing, and Analysis in PCa Disparities Research

Several limitations and recommendations from this review are detailed in Table 3.

*GIS Mapping and Scale Definition:* Almost all studies (24/25) used mapping to visually represent associations between geography and PCa. However, varying geographical scales were adopted, resulting in different findings [41,43,46]. County-level data was most commonly used due to ease of access. However, multiple scales within studies introduced challenges in disentangling personal choice from contextual factors. For example, Meliker et al. observed disappearing survival disparities between NHW and AAs when moving from larger to smaller geographical scales [46]. Oliver et al. detected significant SES associations with PCa outcomes at the census tract level but not at the county level [41]. This phenomenon, known as the Modifiable Areal Unit Problem (MAUP), introduces statistical bias. The recommended geographical scale depends on the research question. Smaller scales might better capture associations with area-level indicators, while larger scales might better detect disparities between geographical areas. To mitigate MAUP, using original point data or smaller units of analysis (e.g., “county” instead of “state”) and performing sensitivity analyses for each geographical scale are suggested [81]. Luo et al. demonstrated the context-dependency of aggregation error using a Monte Carlo simulation, emphasizing the importance of population density consideration [82].

**Figure 6:**
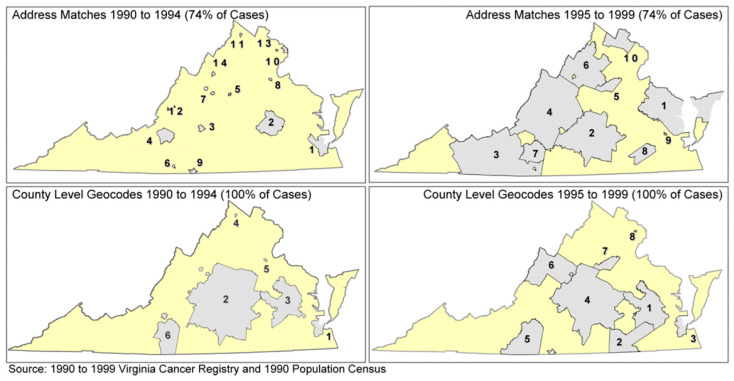
Variation in clusters of PCa incidence based on geocoding quality (Virginia 1990–1999) [84].

*GIS Processing:* Geocoding quality and data smoothing were the main GIS processing applications identified. Only eight studies reported geocoding, with success rates varying between 74% and 100% (Table 1). Standardized geocoding approaches, such as those by NAACCR, are recommended to improve outcome comparability [83]. Insufficient geocoding can lead to systematically missing data and misinforming public health interventions. This was illustrated by Oliver et al., who showed how varying geocoding quality resulted in different cluster formations for PCa patients (Figure 6) [84]. Smoothing techniques help aggregate results of adjacent areas with scarce or missing data but can introduce bias if over-applied. Proper use of smoothing techniques can fill gaps, reduce bias, and prepare data for spatial analysis.

*GIS Analysis:* GIS applications enable rapid spatial analysis of PCa outcomes. Spatial autocorrelation is crucial for examining the impact of space on PCa observations. Three spatial autocorrelation approaches were identified: Global Moran’s I, Tango’s MEET, and Cuzick–Edwards’ k-NN. Global Moran’s I is commonly used to test for global spatial autocorrelation, but Geary’s c test could also be employed [85]. The absence of global spatial autocorrelation does not imply the absence of localized spatial patterns. Cluster detection methods varied, with the Spatial Scan Statistic (SSS), Local Indicator of Spatial Autocorrelation (LISA), and hotspot analysis using the Getis-Ord-Gi statistic being the primary techniques. Variations in SSS model specifications highlight the need for standardization. LISA is more sensitive and specific in cluster detection but increases Type I error with more cases. Hotspot analysis provides color-scaled visual representations of cold and hotspots but is limited by pre-defined geographical boundaries. Combining multiple geospatial approaches, such as hotspot analysis and LISA, is recommended for robust findings. A table summarizing the strengths and weaknesses of the different GIS analysis methods utilized in PCa research is presented below (Table 4).

### 9.5. Future Recommendations for GIS Application in PCa Research and Policy Implications

Future GIS research in PCa disparities should focus on several key areas to enhance the scope and impact of findings.

- Expanding the scope to include treatment and management outcomes is crucial. Utilizing comprehensive databases like SEER-Medicare and SPARCS for procedure-level information will provide valuable insights into healthcare access and utilization, leading to a more holistic understanding of PCa disparities.
- Incorporating both spatial and temporal dimensions in GIS research will allow for a more comprehensive assessment of the cancer burden. This can be achieved through preliminary stratification, joinpoint analysis, or detailed discussions that account for ongoing medical advancements and changes in screening recommendations.
- Ensuring racial inclusivity in study populations is also vital. Future research should extend beyond African Americans (AAs) and non-Hispanic Whites (NHWs) to include other minority groups such as non-Hispanic Asian/Pacific Islanders (NHAPI). This will provide a broader understanding of racial disparities in PCa outcomes.
- Combining multiple geospatial approaches for robust cluster detection and sensitivity analysis will enhance the reliability and validity of research findings. Employing techniques like Spatial Scan Statistic (SSS), Local Indicator of Spatial Autocorrelation (LISA), spatial oblique decision trees (SpODT), and hierarchical Bayesian spatial modeling (HBSM) will offer a comprehensive view of spatial patterns and their underlying causes.
- Addressing geocoding quality and the Modifiable Areal Unit Problem (MAUP) is essential. Researchers should adhere to standardized geocoding principles and report geocoding success rates. Conducting sensitivity analyses across different geographical scales and using original point data when possible will mitigate issues related to MAUP and enhance the robustness of findings.
- Leveraging GIS to identify high-risk regions: GIS mapping has identified specific regions, such as the Mississippi Delta, Appalachia, and parts of the Deep South, with significantly higher PCa mortality and lower survival rates. Continuing to utilize GIS in this aspect has the potential to outline the most deprived areas, in the highest needs of public health interventions.
- Implementing GIS mapping of PCa outcomes for a roadmap toward enhanced healthcare access. Geographical locations of poor PCa outcomes can help deploy mobile screening units and expand telemedicine services to ensure early detection and continuous care for PCa patients in rural and underserved urban areas.
- Addressing Socioeconomic Barriers and implementing financial assistance programs to subsidize the cost of PCa screening, diagnosis, and treatment for low-income populations.
- Launching targeted community-based education and awareness campaigns to inform the public about PCa risks, the importance of early detection, and available healthcare resources.
- Improving Data Collection and Reporting by adopting standardized geocoding methods to enhance the accuracy and comparability of spatial data and facilitate better identification of disparities. It is thus important to foster data sharing between cancer registries, healthcare providers, and public health agencies to support comprehensive analyses and tailored interventions.
- Using GIS mapping to improve travel delays associated with public transportation, especially for minority groups, can enhance PCa care [86]. GIS can identify areas with significant delays, helping optimize transit routes and healthcare facility locations to ensure better access to care.

By addressing these recommendations, future GIS research can leverage spatial analysis to design effective public health interventions, ultimately reducing disparities in PCa outcomes. Including visual aids such as tables and figures can further enhance the clarity of the discussion. For example, a table summarizing the strengths and weaknesses of different GIS methods, a visual representation of geographical scales and their impact on findings, and a flowchart of recommended GIS approaches for PCa disparities research can make the information more digestible. Following these recommendations will ensure that future GIS studies in PCa disparities are more robust, comprehensive, and impactful.

### 9.6. Study Strengths and Limitations

To my knowledge, this is the first systematic review of GIS applications within PCa disparities research. This review is unique as it provided a comprehensive summary of spatial analysis within this disease, highlighted the importance of specific methods in relation to PCa outcomes, and discussed potential gaps while proposing potential solutions. A GIS approach for PCa disparities is crucial for designing efficient and targeted public health interventions. Although this review contains valuable information for future researchers joining the rising trend of GIS research and disparities, a few limitations were encountered. Limitations mainly include the search terms used to select the articles. Some used terms might have been new to the literature, and thus, historical articles describing the same initiative might have been missed by using obsolete terminology. Also, selections have been restricted to published articles only. By doing so, valuable unpublished findings might have been missed, especially since this area of research is evolving rapidly.

## 10. Conclusions

This review highlights current trends in GIScience for PCa surveillance and epidemiology, categorizing GIS approaches into processing, mapping, and analysis. Mapping enables visualization of PCa rates and disparities, processing involves geocoding and rate smoothing, and analysis identifies clusters for public health interventions. Limitations were noted in each area, with recommendations to expand GIS research to address healthcare access disparities, justify scale selections, and combine cluster detection methods for improved accuracy. The review emphasizes interdisciplinary collaboration to enhance PCa disparity studies, guiding future public health and policy interventions effectively.

## Figures and Tables

**Table 1:** Summary of Studies Included in This Systematic Review.

Author (yr)	PCa Database (Period)	Geographic Scale(s)	GIS Application (Method)	Main Outcome(s)	Main GIS Finding(s)
**Jemal A et al. (2002) [38]**	National Center for Health Statistics (1970–1989) *	**County**	**Mapping:** Quantitative and qualitative **Analysis:** Cluster identification (Spatial Scan Statistic)	Disparities in PCa mortality	Five clusters of higher mortality in NHWs and three in AAs. Patterns observed could not be attributed to selected demographic/socioeconomic variables.
**Klassen AC et al. (2005) [39]**	Maryland Cancer Registry (1992–1997)	**Exact patient address, Census block group, County**	**Mapping:** Quantitative and qualitative **Processing:** Geocoding (91%) **Analysis:** Cluster identification (Spatial Scan Statistic)	Disparities in PCa incidence, missing stage, and grade	Six clusters of high/low missing stage and three of missing grade. After adjustment for individual, census block group, and county-level variables, clusters decreased, and patterns changed.
**DeChello LM et al. (2006) [40]**	Connecticut and Massachusetts Tumor Registries (1994–1998) *	**Census tract**	**Mapping:** Quantitative and qualitative **Processing:** Geocoding (NA) **Analysis:** Cluster identification (Spatial Scan Statistic)	Disparities in PCa incidence	Significant high and low clusters for both NHW and AA men identified. In NHWs, higher incidence clusters had higher census-tract SES. Differences in race-specific geographic distribution of incidence do not suggest a shared environmental etiology.
**Oliver M N et al. (2006) [41]**	Virginia Cancer Registry (1990–1999) *	**Census tract** **County**	**Mapping:** Quantitative and qualitative **Processing:** Geocoding (74%–100%) and smoothing (headbanging) **Analysis:** Spatial autocorrelation (MEET), cluster identification (Spatial Scan Statistic)	Disparities in PCa incidence	Significant overall clustering with elevated incidence in eastern and central locations.
**Gregorio DI et al. (2007) [42]**	Connecticut Tumor Registry (1984–1998)	**Exact patient address**	**Mapping:** Qualitative **Analysis:** Cluster identification (Spatial Scan Statistic)	Disparities in PCa survival	Identification of three geographical clusters. Adjusting for age, tumor grade, stage, and race reduced clusters to one. PCa survival varies, only in part, according to place of residence.
**Xiao H et al. (2007) [43]**	Florida Cancer Data System (1990–2001) *	**Census tract** **County**	**Mapping:** Quantitative **Processing:** Geocoding (NA)	Disparities in PCa incidence, stage, and grade	Maps showing greatest racial disparities in incidence and late-stage PCa in the northern and central counties.
**Hsu C E et al. (2007) [44]**	Texas prostate cancer-specific death cases file (1980–2001)	**County**	**Mapping:** Qualitative **Analysis:** Cluster identification (Spatial Scan Statistic)	Disparities PCa mortality	Identification of statistically significant geographic counties with excess mortality rates for each of the racial groups studied and examination of those trends in function of time.
**Hinrichsen VL (2009) [45]**	Maryland Cancer Registry (1992–1997)	**Census block groups**	**Processing:** Geocoding (NA) **Analysis:** Spatial autocorrelation (Cuzick–Edwards’ k-NN, Global Moran’s I, MEET)	Disparities PCa stage and grade	For both grade and stage at diagnosis, Cuzick–Edwards’ k-NN and Moran’s I were very sensitive to the % of pop. parameter. For stage, all three tests showed that adjusting for individual and area level variables reduced clustering, but not entirely.
**Meliker JR et al. (2009) [46]**	Michigan Cancer Surveillance Program (1985–2002)	**FHLD, SHLD** **Neighborhoods**	**Mapping:** Quantitative **Processing:** Geocoding (91%)	Disparities in PCa survival	NHWs had significantly higher survival rates compared with AAs at the FHLD; however, in smaller geographic units (SHLD, neighborhoods), disparities diminished and disappeared.
**Hébert JR (2010) [47]**	South Carolina Cancer Registry (2001–2005)	**DHEC Region**	**Mapping:** Quantitative **Processing:** Geocoding (82%–100%)	Disparities in PCa MIR	Striking differences in MIR mapping between AAs and NHWs in the 8 DHEC regions examined.
**Altekruse et al. (2010) [48]**	State cancer registries of Tennessee, Alabama, Georgia, and Florida (1999–2001) *	**Census tract**	**Mapping:** Qualitative **Analysis:** Cluster identification (Spatial Scan Statistic)	Disparities in PCa incidence (localized)	Identification of statistically significant clusters. Higher incidence of localized disease in urban areas.
**Goovaerts P et al. (2011) [49]**	Florida Cancer Data System (1981–2007)	**County**	**Mapping:** Quantitative and qualitative **Processing:** Smoothing (Binomial Kriging)	Disparities in late stage PCa	Recent increase in the frequency of late-stage diagnosis in urban areas. The annual rate of decrease in late-stage diagnosis and the onset years for significant declines varied greatly among counties and racial groups.
**Xiao H et al. (2011) [50]**	Florida Cancer Data System (1996–2002) *	**Census tract** **County**	**Mapping:** Quantitative and qualitative **Processing:** Geocoding (NA), smoothing (Binomial Kriging)	Disparities in late-stage PCa	More counties had higher rates of late-stage diagnosis for AA men than for NHW men, and the location of these racial disparities changed with time.
**Goovaerts P et al. (2012) [51]**	Florida Cancer Data System (1981–2007)	**County**	**Mapping:** Quantitative and qualitative **Processing:** Smoothing (Binomial Kriging) **Analysis:** Cluster identification (spatially weighted cluster analysis)	Disparities in late-stage PCa	Geographical disparities were most widespread upon introduction of PSA screening. Spatially weighted cluster analysis resulted in spatially compact groups of counties with similar temporal trends.
**Goovaerts P (2013) [52]**	Florida Cancer Data System (1981–2007)	**County**	**Mapping:** Quantitative and qualitative **Processing:** Smoothing (Binomial Kriging) **Analysis:** Cluster identification (spatially weighted cluster analysis)	Disparities in late-stage PCa	A temporal trend in late-stage diagnosis suggests the existence of geographical disparities in the implementation and/or impact of the newly introduced PSA screening.
**Wagner S et al. (2013) [53]**	Georgia Comprehensive Cancer Registry (1998–2008)	**Census tract** **County**	**Mapping:** Quantitative and qualitative **Analysis:** Cluster identification (Getis-Ord-Gi and Spatial Scan Statistic)	Disparities in incidence and high grade or stage PCa	Pattern of higher incidence and more advanced disease found in northern and northwest central Georgia. Hotspot revealed six significant clusters of higher incidence for both races. When stratified by race, clusters among NHW and AA men were similar, although centroids were slightly shifted.
**Gregorio DI (2013) [54]**	Connecticut Tumor Registry (1994–1998)	**Exact patient address**	**Mapping:** Qualitative **Analysis:** Cluster identification (Spatial Scan Statistic)	PCa incidence	Two locations where incidence rates significantly exceeded the statewide level and two locations with significantly lower disease rates. Analysis adjusted for age and covariation of colorectal cancer incidence rates across the state accounted for all significant variations previously observed.
**Goovaerts P (2015) [55]**	Florida Cancer Data System (2001–2007) *	**Census tract** **County**	**Mapping:** Quantitative and qualitative **Analysis:** Geographically Weighted Regression	Disparities in late-stage PCa	Identification of locations where ORs for late-stage are higher/lower than the state level.
**Wang M et al. (2017) [56]**	Pennsylvania Cancer Registry (2000–2011) *	**County**	**Mapping:** Quantitative and qualitative **Processing:** Smoothing (Empirical Bayes) **Analysis:** Spatial autocorrelation (Global Moran’s I), cluster identification (Local Moran’s I)	Disparities in PCa incidence	Incidence of PCa among NHW males declined from 2000–2002 to 2009–2011, with significant variation across geographic regions.
**Wang, M et al. (2020) [57]**	Pennsylvania Cancer Registry (2004–2014)	**Exact patient address**	**Mapping:** Quantitative mapping **Processing:** Smoothing (Inverse Distance Weighting)	Disparities in aggressive PCa	Counties where AA population is lower than 5.3% have the highest odds of having the most aggressive forms of PCa in those AA men
**Aghdam et al. (2020) [58]**	Single institutional database (2008–2017) *	**Zip code**	**Mapping:** Qualitative	Disparities in PCa management	Travel distance did not prevent the uptake of SBRT for African American, elderly, or rural patients.
**Georgantopoulos, P. et al. (2021) [59]**	US Veterans Health Administration EMR (1999–2015)	**ZCTA**	**Mapping:** Quantitative and qualitative **Analysis:** Spatial autocorrelation (Global Moran’s I), cluster identification (Local Moran’s I)	Disparities in PCa MIR	Identification of spatial clusters of higher- or lower-than-expected MIRs by ZCTA. Two clusters of higher-than-expected MIRs were found in the upstate region.
**Moore J. X. et al. (2022) [60]**	CDC (1999–2019)	**County**	**Mapping:** Qualitative **Processing:** Smoothing (Empirical Bayes) **Analysis:** Cluster identification (Getis-Ord-Gi and Local Moran’s I)	Disparities in PCa mortality	Cancer mortality hotspots were heavily concentrated in three major areas in Georgia. Hotspot counties generally had a higher proportion of AA adults, older adult population, greater poverty, and more rurality
**Aladuwaka et al. (2022) [61]**	Alabama State Cancer Profile data (NA) *	**County**	**Mapping:** Quantitative and qualitative	Disparities in PCa incidence and mortality	Apparent socioeconomic disparity between the AA Belt and non-AA Belt counties of Alabama, which suggests that disparities in PCa incidence and mortality are strongly related to SES.
**Tang C. et al. (2021) [62]**	National Medicare Database (2011–2014)	**Zip code** **County**	**Mapping:** Quantitative and qualitative	Disparities in PCa management	Patient access was most limited for brachytherapy. Lower provider availability in rural areas, especially in western states. Heterogeneity in the access of definitive PCa treatment. Greater distance was associated with a decreased probability of treatment.

* PCa database linked to census data.

**Table 2:** Variations in the Spatial Scan Statistic Technique for Cluster Identification.

Study	Scanning Window Shape	Scanning Window Size	Clusters Delimited by Geopolitical Boundaries	Outcome
**Jemal A et al. (2002) [38]**	Circular	0–50% of the total population at risk.	Yes (county)	PCa mortality
**Klassen AC et al. (2005) [39]**	Circular	0–50% of the total population at risk.	No	PCa incidence, missing stage, and grade
**DeChello LM et al. (2006) [40]**	Circular	0–50% of the total population at risk.	No	PCa incidence
**Oliver M N et al. (2006) [41]**	NA	NA (A Spatial Scan Statistic was used to evaluate raw counts).	NA (clusters not mapped)	PCa incidence
**Gregorio DI et al. (2007) [42]**	Circular	NA (varying sizes across the geography of the study area).	No	PCa survival
**Hsu et al. (2007) [44]**	NA	50% and 90% of the study period. 50% of the population at risk.	Yes (county)	PCa mortality
**Altekruse et al. (2010) [48]**	Circular and elliptical	0–50% of the total population at risk.	Yes (census tract)	PCa incidence (localized)
**Wagner S et al. (2013) [53]**	Circular	50% spatial scanning window.	No	Incidence and high-grade or stage PCa
**Gregorio DI (2013) [54]**	Circular	NA (scanning circles at random locations and of varying sizes).	No	PCa incidence

NA = Not Applicable.

**Table 3:** Summary of GIS Applications, Limitations, and Proposed Recommendations in PCa Research.

GIS Application	Limitation(s)/Gap(s)	Proposed Recommendations(s)
**Overall Scope**	Limited focus on PCa management and/or treatment Limited variability in PCa database types Limited focus on racial disparities in remaining minority groups (main focus on NHWs and AAs)	Include more GIS research on PCa and procedure utilization in PCa patients (i.e., access to screening) Utilize claims databases for procedure information Include the temporal element to account for clinical advancement in PCa procedures and changes in guidelines Include other racial categories that have proven to exhibit PCa disparities (i.e., NHAPI, NHAIAN)
**Mapping**	Lack of justification for the determination of geographic scale for PCa inferences Varying PCa associations dependent on the geographical scale adopted (MAUP)	Consider larger scales for examining PCa disparities in-between geographical locations Consider smaller scales when examining associations between PCa outcomes and area-level characteristics Utilize original point data instead of aggregates if possible Create districts based on the spatial patterns observed in the selected PCa dataset Include sensitivity analysis across different geographical scales
**Processing**	Low-quality geocoding leading to inaccurate PCa cluster detection Over-smoothing	Adhere to geocoding principles as per NAACR Always include the geocoding quality percentage Avoid over-smoothing and utilize imputation techniques for missing PCa data as appropriate
**Analysis**	Lack of initial global spatial autocorrelation testing Variability in cluster detection methods, especially when using the Spatial Scan Statistic	Always include global spatial autocorrelation as an initial step to assess for overall dispersion in PCa outcomes Employ alternative cluster detection methods that exhibit less variability (i.e., LISA) or have proven to be superior in cluster detection (i.e., SpODT and HBSM) Combine cluster detection techniques for more robust and comprehensive findings (i.e., hotspot analysis followed by SSS or LISA)

NHAPI: Non-Hispanic Asian Pacific Islander, NHAIAN: Non-Hispanic American Indian/Alaskan Native, MAUP: Modifiable Areal Unit Problem, LISA: Local Indicator of Spatial Autocorrelation, SpODT: Spatial oblique decision tree, HBSM: Hierarchical Bayesian spatial modeling.

**Table 4:** Summary of strengths and weaknesses of GIS analysis methods applied in PCa research.

Method	Strengths	Weaknesses	Example of Recommended Application
**Spatial Scan Statistic (SSS)**	Provides the location, size, and statistical significance of PCa clusters Identifies areas with higher-than-expected PCa rates Publicly available	Sensitivity to parameters: the choice of scanning window, shape, and size can influence the results Assumes circular or elliptical cluster shapes Computational complexity increases with larger datasets	To detect significant circular or elliptical clusters of high PCa mortality within a specific region, accounting for the population at risk and considering varying cluster sizes
**Local Moran’s I (LISA)**	Identifies areas where PCa cases are spatially clustered or dispersed Does not need a priori specification of a scan window shape and size More appropriate for finer scales (census tracts, neighborhoods)	Higher probability of false positives with an increasing number of cases Scale sensitivity	To identify statistically significant clusters of high or low PCa incidence rates, provide insight into neighboring observations, and understand spatial patterns of PCa incidence at smaller scales (census tracts, neighborhoods)
**Hotspot Analysis (Getis-Ord Gi statistic)**	Allows for the visual identification of geographically-delimited clusters at the local level (i.e., census, county) Helps to pinpoint geographical-limited areas with high or low prostate cancer rates	Identified areas are limited by geopolitical boundaries Scale sensitivity	To identify local hotspots or coldspots of PCa incidence within a specific geographic area, such as a county or a census tract
**Geographically Weighted Regression (GWR)**	Recognizes spatially varying relationships Allows for localized and more accurate modeling of the relationships between variables Captures spatial heterogeneity Aids in the identification of localized clusters or spatial patterns of PCa outcomes	May require a relatively large sample size to ensure reliable estimation and avoid issues of spatial outliers or sparse data in specific regions Increased computational requirements (estimates regression coefficients for each location) Requires understanding of the spatial context for accurate interpretation Multicollinearity	To investigate the locally dynamic relationship between area-level characteristics (e.g., racial composition, socioeconomic status, availability of healthcare) and PCa outcomes (i.e., appropriate for multilevel analyses)

## Data Availability

The full search strategy is included in Appendix A and can be applied for future reproducibility.

## Appendix A. Research Strategy

**Research Question**		
**State Question: What are the different geospatial approaches for quantifying health disparities in prostate cancer outcomes?** **Specific Inclusions/Exclusions:** **Inclusion:** Studies that examine disparities in PCa using geographical elements as independent variables **Exclusion:** Studies conducted outside the US, studies that did not assess for a direct relationship between a geographical component and PCa disparities were excluded.	**Select Core Databases:** PubMed Embase Web of Science	**Limits:** English only Years: Up to 2022 Age Groups: Adults 18 years and older

	**Concept: Health Disparities**	**Concept: Geospatial Analysis**	**Concept: Prostate Cancer**
Thesaurus Terms/Subheadings	“Socioeconomic Factors”[Mesh] OR “Health Status Disparities”[Mesh] OR “Healthcare Disparities”[Mesh] OR “Health Services Accessibility”[Mesh] OR “Vulnerable Populations”[Mesh] OR	“Geospatial analysis”[MeSH Terms] OR “geographic [MeSH]) OR (geographical[MeSH]) OR (spatial[MeSH])	“Prostatic Neoplasms”[Mesh] OR
Textwords	socioeconomic* OR disparit* OR vulnerable OR “healthcare access” OR “healthcare accessibility” OR “health service accessibility” OR “health services accessibility”	Geograph* OR Spatial OR Geospatial OR GIS OR Place of Residence OR Mapping	“prostate cancer” OR “prostate cancers” OR “cancer of the prostate” OR “prostatic neoplasms” OR “prostate neoplasms”

**PubMED SEARCH STRATEGIES**

**Search Number**	**Query**	**Search Details**	**Results**
4	(((“Prostatic Neoplasms”[Mesh] OR “prostate cancer” OR “prostate cancers” OR “cancer of the prostate” OR “prostatic neoplasms” OR “prostate neoplasms”)) AND ((“Socioeconomic Factors”[Mesh] OR “Health Status Disparities”[Mesh] OR “Healthcare Disparities”[Mesh] OR “Health Services Accessibility”[Mesh] OR “Vulnerable Populations”[Mesh] OR socioeconomic* OR disparit* OR vulnerable OR “healthcare access” OR “healthcare accessibility” OR “health service accessibility” OR “health services accessibility”)))) AND (“Geography”[Mesh] OR “Geography, Medical”[Mesh] OR geograph* OR spatial OR geospatial* OR geospatial analysis OR GIS OR Mapping OR “Place of Residence”) Filters: English	(“Prostatic Neoplasms”[MeSH Terms] OR “prostate cancer”[All Fields] OR “prostate cancers”[All Fields] OR “cancer of the prostate”[All Fields] OR “Prostatic Neoplasms”[All Fields] OR “prostate neoplasms”[All Fields]) AND (“Socioeconomic Factors”[MeSH Terms] OR “Health Status Disparities”[MeSH Terms] OR “Healthcare Disparities”[MeSH Terms] OR “Health Services Accessibility”[MeSH Terms] OR “Vulnerable Populations”[MeSH Terms] OR “socioeconomic*”[All Fields] OR “disparit*”[All Fields] OR (“vulnerabilities”[All Fields] OR “vulnerability”[All Fields] OR “vulnerable”[All Fields] OR “vulnerables”[All Fields]) OR “healthcare access”[All Fields] OR “healthcare accessibility”[All Fields] OR “health service accessibility”[All Fields] OR “Health Services Accessibility”[All Fields]) AND (“Geography”[MeSH Terms] OR “geography, medical”[MeSH Terms] OR “geograph*”[All Fields] OR (“spatial”[All Fields] OR “spatialization”[All Fields] OR “spatializations”[All Fields] OR “spatialized”[All Fields] OR “spatially”[All Fields]) OR “geospatial*”[All Fields] OR ((“geospatial”[All Fields] OR “geospatially”[All Fields]) AND (“analysis”[MeSH Subheading] OR “analysis”[All Fields])) OR (“proc acm sigspatial int conf adv inf”[Journal] OR “gis”[All Fields]) OR (“mapped”[All Fields] OR “mapping”[All Fields] OR “mappings”[All Fields]) OR “Place of Residence”[All Fields])	320
3	“Geography”[Mesh] OR “Geography, Medical”[Mesh] OR geograph* OR spatial OR geospatial* OR geospatial analysis OR GIS OR Mapping OR “Place of Residence”	“Geography”[MeSH Terms] OR “geography, medical”[MeSH Terms] OR “geograph*”[All Fields] OR (“spatial”[All Fields] OR “spatialization”[All Fields] OR “spatializations”[All Fields] OR “spatialized”[All Fields] OR “spatially”[All Fields]) OR “geospatial*”[All Fields] OR ((“geospatial”[All Fields] OR “geospatially”[All Fields]) AND (“analysis”[MeSH Subheading] OR “analysis”[All Fields])) OR (“proc acm sigspatial int conf adv inf”[Journal] OR “gis”[All Fields]) OR (“mapped”[All Fields] OR “mapping”[All Fields] OR “mappings”[All Fields]) OR “Place of Residence”[All Fields]	1,116,497
2	(“Socioeconomic Factors”[Mesh] OR “Health Status Disparities”[Mesh] OR “Healthcare Disparities”[Mesh] OR “Health Services Accessibility”[Mesh] OR “Vulnerable Populations”[Mesh] OR socioeconomic* OR disparit* OR vulnerable OR “healthcare access” OR “healthcare accessibility” OR “health service accessibility” OR “health services accessibility”))	“Socioeconomic Factors”[MeSH Terms] OR “Health Status Disparities”[MeSH Terms] OR “Healthcare Disparities”[MeSH Terms] OR “Health Services Accessibility”[MeSH Terms] OR “Vulnerable Populations”[MeSH Terms] OR “socioeconomic*”[All Fields] OR “disparit*”[All Fields] OR “vulnerabilities”[All Fields] OR “vulnerability”[All Fields] OR “vulnerable”[All Fields] OR “vulnerables”[All Fields] OR “healthcare access”[All Fields] OR “healthcare accessibility”[All Fields] OR “health service accessibility”[All Fields] OR “Health Services Accessibility”[All Fields]	950,029
1	(“Prostatic Neoplasms”[Mesh] OR “prostate cancer” OR “prostate cancers” OR “cancer of the prostate” OR “prostatic neoplasms” OR “prostate neoplasms”)	“Prostatic Neoplasms”[MeSH Terms] OR “prostate cancer”[All Fields] OR “prostate cancers”[All Fields] OR “cancer of the prostate”[All Fields] OR “Prostatic Neoplasms”[All Fields] OR “prostate neoplasms”[All Fields]	184,831

**EMBASE SEARCH STRATEGIES**

**No.**	**Query**	**Results**
#4	#1 AND #2 AND #3	317
#3	(‘geography’ OR ‘geography, medical’ OR geograph* OR spatial OR geospatial* OR geospatial) AND analysis OR gis OR mapping OR ‘place of residence’	682,890
#2	‘socioeconomic factors’ OR ‘health status disparities’ OR ‘healthcare disparities’ OR ‘vulnerable populations’ OR socioeconomic* OR disparit* OR vulnerable OR ‘healthcare access’ OR ‘healthcare accessibility’ OR ‘health service accessibility’ OR ‘health services accessibility’	583,093
#1	‘prostatic neoplasms’/exp OR ‘prostatic neoplasms’	291,595

**WEB OF SCIENCE SEARCH STRATEGIES**

**Query**	**Results**
(‘prostatic neoplasms’ OR ‘prostatic neoplasms’) AND (‘socioeconomic factors’ OR ‘health status disparities’ OR ‘healthcare disparities’ OR ‘vulnerable populations’ OR socioeconomic* OR disparit* OR vulnerable OR ‘healthcare access’ OR ‘healthcare accessibility’ OR ‘health service accessibility’ OR ‘health services accessibility’) AND ((‘geography’ OR ‘geography, medical’ OR geograph* OR spatial OR geospatial* OR geospatial) AND analysis OR gis OR mapping OR ‘place of residence’)	16
((‘geography’ OR ‘geography, medical’ OR geograph* OR spatial OR geospatial* OR geospatial) AND analysis OR gis OR mapping OR ‘place of residence’)	3,173,703
(‘socioeconomic factors’ OR ‘health status disparities’ OR ‘healthcare disparities’ OR ‘vulnerable populations’ OR socioeconomic* OR disparit* OR vulnerable OR ‘healthcare access’ OR ‘healthcare accessibility’ OR ‘health service accessibility’ OR ‘health services accessibility’)	629,895
(‘prostatic neoplasms’ OR ‘prostatic neoplasms’)	8363
